# miR-154-3p and miR-487-3p synergistically modulate RHOA signaling in the carcinogenesis of thyroid cancer

**DOI:** 10.1042/BSR20193158

**Published:** 2020-01-02

**Authors:** Xiang-da Fan, Yan Luo, Jun Wang, Ning An

**Affiliations:** 1 Department of Head and Neck Oncology, Gansu Provincial Cancer Hospital and Gansu Provincial Academic Institute for Medical Research, Lanzhou 730050, China; 2 Department of Pathology, Gansu Provincial Cancer Hospital and Gansu Provincial Academic Institute for Medical Research, Lanzhou 730050, China

**Keywords:** miR-154-3p, miR-487-3p, RHOA, synergistic effect, thyroid cancer

## Abstract

Background: miRs family members are often thought to have extensively overlapping targets and synergistically to modulate target gene expression via post-transcriptional repression. The present study was to determine whether miR-154-3p and miR-487-3p synergistically collaborated to regulate RHOA signaling in the carcinogenesis of thyroid cancer. Materials and methods: Candidate miRs were filtrated using miR microarray assays. Gene and protein expression levels were analyzed using RT-qPCR and Western blotting, respectively. Cell growth was evaluated using CCK8 assays and nude-mouse transplanted tumor experiments. Cell apoptosis was detected using Annexin V-FITC double staining. Results: miR-154-3p and miR-487-3p were significantly decreased in 63 thyroid cancer tissues and cell lines compared with those in paired non-tumor tissues and normal thyroid follicular epithelial cells. Low expression levels of miR-154-3p and miR-487-3p significantly correlated with tumor size, TNM stage, histological grade, lymph node metastasis and shorter overall survival in patients with thyroid cancer. Furthermore, the protein expression of RHOA was significantly inversely correlated with miR-154-3p (*r* = −0.404; *P* = 0.001) and miR-487-3p (*r* = −0.456; *P* < 0.001) expression in thyroid cancer tissues. We experimentally validated that miR-154-3p and miR-487-3p synergistically blocked thyroid cancer cell growth *in vitro* and *in vivo*. However, the anti-proliferative and pro-apoptotic activities of miR-154-3p/487-3p were neutralized by RHOA overexpressed vectors. Conclusions: Our present findings expounded a novel signal cascade employing miR-154-3p/487-3p and RHOA to fine-tune thyroid cancer cell proliferation and apoptosis. We corroborated that suppression of RHOA by miR-154-3p/487-3p may be a valuable therapeutic target for impeding thyroid cancer progression.

## Introduction

Thyroid cancer as a common endocrine cancer ranks ninth for incidence worldwide [1]. Global cancer statistics in 2018 estimates that approximately 567,000 cases are diagnosed with thyroid cancer [1]. Among these cancer patients, about 15.9% cases originate from China, and the incidence rate in women is three times higher than in men [2,3]. Recently, the incidence of thyroid cancer is ascending, which may be attributed to the improvement of the early diagnosis of thyroid cancer [4]. Although the therapeutic strategies, including thyroidectomy, radioiodine therapy and thyroid-stimulating hormone inhibition therapy, have greatly improved the survival quality of early thyroid cancer patients, the prognosis of advanced and metastatic patients is dissatisfactory [5]. Therefore, it is very meaningful to investigate the underlying molecular mechanism for providing the meritorious therapeutic protocol of thyroid cancer patients.

Ras homolog family member A (RHOA) as a representative protein of RHO subfamily primarily participates in actin cytoskeleton rearrangement, which is implicated in various physiological functions, including cell proliferation, differentiation, migration, polarity and gene transcription [6]. Rho-associated protein kinase (ROCK) is a downstream effector of RHOA and has the ability to modulate cytoskeleton dynamics [6]. Over-activation of RHOA/ROCK signaling pathway may be associated with a decrease in cell–cell junctions and a promotion of cell motility [7]. Previous studies have validated that high expression of RHOA and ROCK is frequently reported in various cancer-types, such as breast cancer, ovarian cancer and colon cancer [6,8,9]. In thyroid cancer, RHOA/ROCK signaling has a profound stimulative impact to facilitate the invasiveness of anaplastic thyroid cancer cells [10].

microRNAs (miRs) are a class of non-coding RNAs and have widely been elaborated in the pathogenesis of various diseases, including carcinogenesis [11]. miRs function as post-transcriptional regulators to repress target genes translation via binding with their 3′-untranslated regions (3′-UTRs) [12]. Numerous miRs are deregulated in thyroid cancer tissues and perform tumor-suppressive or oncogenic roles in thyroid carcinoma [13,14]. For example, oncogene ROCK1 can be suppressed by miR-584 to block migration and invasion in thyroid carcinoma [14]. However, the roles of miR-154-3p and miR-487-3p have not been clarified in the progression of thyroid carcinoma. In the present study, a significant decrease in miR-154-3p and miR-487-3p expression was observed in thyroid carcinoma specimens compared with adjacent non-tumor tissues. Moreover, the molecular mechanism underlying in miR-154-3p- and miR-487-3p-induced thyroid carcinoma cell growth inhibition and apoptosis was investigated, and the results demonstrated that RHOA/ROCK signaling was implicated in the tumor-suppressive roles of miR-154-3p and miR-487-3p.

## Materials and methods

### Human specimens

Sixty-three pairs of thyroid cancer tissues and adjacent non-tumor tissues were collected from thyroid cancer patients who had undergone surgery. All samples were immediately stored in an ultra-low temperature refrigerator. Signed informed consent forms were obtained from all thyroid cancer patients. The study was approved by the Ethics Committee of the Gansu Provincial Cancer Hospital & Gansu Provincial Academic Institute for Medical Research.

### Cell culture

A normal thyroid follicular epithelium cell line (Nthy-ori3-1), three papillary thyroid carcinoma cell lines (K-1, TPC-1 and B-CPAP) and anaplastic thyroid cancer cell lines (CAL-62 and 8305C) were obtained from the American Type Culture Collection (ATCC). Nthy-ori3-1 cells were cultured in CM-H023 medium (Procell, China), and thyroid cancer cell lines were cultured in RPMI-1640 medium (Life Technologies, Carlsbad, CA, U.S.A.) supplemented with penicillin G (100 U/ml), streptomycin (100 mg/ml) and 10% fetal bovine serum (FBS, Life Technologies) with 5% CO_2_ atmosphere at 37°C.

### Cell Counting Kit-8 (CCK8) assay

Cell proliferation was measured using CCK8 (DojinDO, Japan) assays every 24 h for three days. Transfected cells (1 × 10^4^ cells/well) were seeded into 96-well plates and cultured at 37°C with 5% CO_2_. After incubated with CCK8 (10 μl) for 2 h, the absorbance was measured at 450 nm a spectrophotometer (Thermo Fisher, U.S.A.).

### Flow cytometry for apoptosis

Annexin V-FITC/PI apoptosis detection kit was purchased from Invitrogen (Carlsbad, Calif, U.S.A.). The apoptosis of transfected cells was performed by flow cytometry analysis (FACScan, BD Biosciences, San Jose, CA, U.S.A.). The apoptotic cell proportion was analyzed by CELL Quest 3.0 software (BD Biosciences).

### Microarray assays

Microarray analysis was performed using Agilent Human miRNA (8*15K) V14.0 arrays of Ribobio (Guangzhou, China), as described previously [15]. miRs expression profiling was detected using miR microarray assays (*n* = 3 in each group). miRNeasy Mini Kit (Qiagen, Inc., Valencia, CA, U.S.A.) was used to extract total RNA, according to the manufacturer’s protocol. miRs were labeled with Hy3 or Hy5 fluorescence using the miRCURY™ Array Power Labeling Kit (Exiqon) to obtain the fluorescent probe that can be hybridized with the chip. The labeled probe was hybridized with the miRCURY™ chip under the standard condition using the MAUI hybridization system. Agilent scanner and the Feature Extraction 10.7.1.1 software (Agilent Technologies) were used to obtain the microarray raw data. Microarray results were analyzed using the GeneSpring GX 12.5 software (Agilent Technologies). Differentially expressed miRs were selected out according to |Log2fold change| ≥ 1, *P* < 0.05 and false discovery rate < 0.05. The hierarchical clustering analysis was performed using MeV software (version 4.2.6).

### Reverse transcription-quantitative polymerase chain reaction (RT-qPCR)

RT-qPCR for miRs: total RNA was extracted using miRNeasy Mini Kit (Qiagen, Inc., Valencia, CA, U.S.A.), according to the manufacturer’s protocol. TaqMan® RT kit and TaqMan® MicroRNA assay (Applied Biosystems) were used to detect miRs expression levels using Applied Biosystems 7300 Real-Time PCR System (Thermo Fisher Scientific, Inc.). miRs expression levels were calculated using 2^−ΔΔ*C*^_t_ method, as described previously [16], and U6 was used as an internal control.

RT-qPCR for mRNA: Moloney murine leukemia virus reverse transcriptase (Invitrogen) was used to synthesize cDNA with 2 μg of total RNA according to the manufacturer’s protocol. Real-Time PCR was performed using Applied Biosystems 7300 System with the TaqMan Universal PCR Master Mix (Thermo Fisher Scientific). The relative expression levels of RHOA were calculated using the 2^−ΔΔ*C*^_t_ method [16], and glyceraldehyde-3-phosphate dehydrogenase (GAPDH) was served as the internal control. The primers were used as follows: RHOA: Forward 5′-AGCCTGTGGAAAGACATGCTT-3′ and Reverse 5′-TCAAACACTGTGGGCACATAC-3′; GAPDH: forward 5′-GCACCGTCAAGCTGAGAAC-3′ and reverse 5′-TGGTGAAGACGCCAGTGGA-3′.

### Western blotting

Western blotting procedures were performed as previously described [17]. The primary antibody for RHOA (cat. no: ab86297; dilution: 1: 1000) and ROCK1 (cat. no: ab45171; dilution: 1: 1000) were purchased from Abcam (Cambridge, U.K.). Horseradish peroxidase-conjugated secondary antibody was obtained from Santa Cruz Biotechnology, Inc. (Dallas, TX, U.S.A.). Protein bands were visualized using an enhanced chemiluminescence kit (Thermo Fisher Scientific, Inc.). Signals were analyzed with Quantity One® software version 4.5 (Bio Rad Laboratories, Inc., Hercules, CA, U.S.A.). Anti-β-actin (cat. no. sc-130065; dilution: 1: 2000; Santa Cruz Biotechnology) was used to as the control antibody.

### Immunohistochemical (IHC) staining

The paraffin-embedded tumor tissues and adjacent non-tumor tissues were cut into 3-μm sections and mounted on glass slides for staining with immunoperoxidase, and the procedures of immunohistochemical staining of RHOA (cat. no: ab86297; dilution: 1: 100; Abcam, Cambridge, U.K.) and ROCK1 (cat. no: ab45171; dilution: 1: 100) were performed as described previously [18]. The pictures were visual under a microscope (Leica DM 2500; Leica Microsystems GmbH, Wetzlar, Germany). Image Pro-Plus 6 software (Media Cybernetics, Inc., Rockville, MD, U.S.A.) was used for the analysis of the integrated optical density of positive-RHOA and -ROCK1.

### Cell transfection and vector constructs

Pre-miR-control (pre-miR-Con), pre-miR-154-3p and pre-miR-487-3p were purchased from Guangzhou RiboBio Co., Ltd. (Guangzhou, China) and transfected into thyroid cancer cells using Lipofectamine 2000 (Invitrogen) according to the manufacturer’s protocols. RHOA overexpressed plasmids were purchased from GeneCopoeia, Inc. (Rockville, MD, U.S.A.). RHOA overexpressed vectors were transfected into BCa cells using Lipofectamine 2000 (Invitrogen), according to the manufacturer’s protocols.

### Luciferase reporter assay

RHOA with wild-type (WT) and mutant-type (MUT) 3′-UTR were synthesized by Sangon (Shanghai, China) and inserted into the pmirGLO Dual-Luciferase miRNA Target Expression Vector (Promega). For the luciferase assay, thyroid cancer cells (1 × 10^5^) were seeded into 24-wells and co-transfected with luciferase reporter vectors containing WT or MUT 3′-UTR (0.5 μg) of RHOA combined with pre-miR-Con, pre-miR-154-3p, pre-miR-487-3p or pre-miR-154-3p/487-3p (100 nM) using Lipofectamine 2000 (Invitrogen; Thermo Fisher Scientific, Inc.) at 37˚C for 48 h. Luciferase activity was measured using a dual luciferase reporter assay kit (Beyotime Institute of Biotechnology), according to the manufacturer’s protocol.

### Tumor formation assay *in vivo*

Human thyroid cancer K-1 cells stably transfected with pre-miR-Con, pre-miR-154-3p, pre-miR-487-3p or pre-miR-154-3p/487-3p, and K-1 were suspended in phosphate-buffered saline (PBS) and injected subcutaneously into the same side armpit of each nude mouse (1 × 10^7^ cells per 0.1 ml). Four-week-old male BALB/c nude mice were purchased from (*n* = 24, Beijing HFK Bio-Technology. co., LTD., Beijing, China). Animal experiment was performed in the experimental center of the Gansu Provincial Cancer Hospital & Gansu Provincial Academic Institute for Medical Research, Lanzhou, China. Tumor weight was measured when mice were killed on week 4 by intraperitoneal injection of sodium pentobarbital (2%; 200 mg/kg; cat. no. P3761; Sigma-Aldrich; Merck Millipore, Germany). All protocols were approved by the Animal Care and Research Committee of the Gansu Provincial Cancer Hospital & Gansu Provincial Academic Institute for Medical Research, Lanzhou, China (Approval number: 2018030155).

### Statistical analysis

Data were presented as mean ± SD. Statistical analysis was performed using GraphPad Prism Version 7.0 (GraphPad Software, Inc., La Jolla, CA, U.S.A.). Chi-Square (χ^2^) tests were used to evaluate differences between the clinical characteristics and miRs expression. Student *t*-test was used to analyze two-group differences. Inter-group differences were analyzed by one-way analysis of variance, followed by Tukey’s post hoc analysis. Survival analysis was performed using the Kaplan–Meier method with the log-rank test applied for comparison. Spearman’s rank analysis was used to identify the correlation between the expression levels of RHOA and miR-154-3p or miR-487-3p in thyroid cancer tissues. *P* < 0.05 was considered to indicate a statistically significant difference.

## Results

### miR-154-3p and miR-487-3p are down-regulated in thyroid cancer tissues

**Figure 1:**
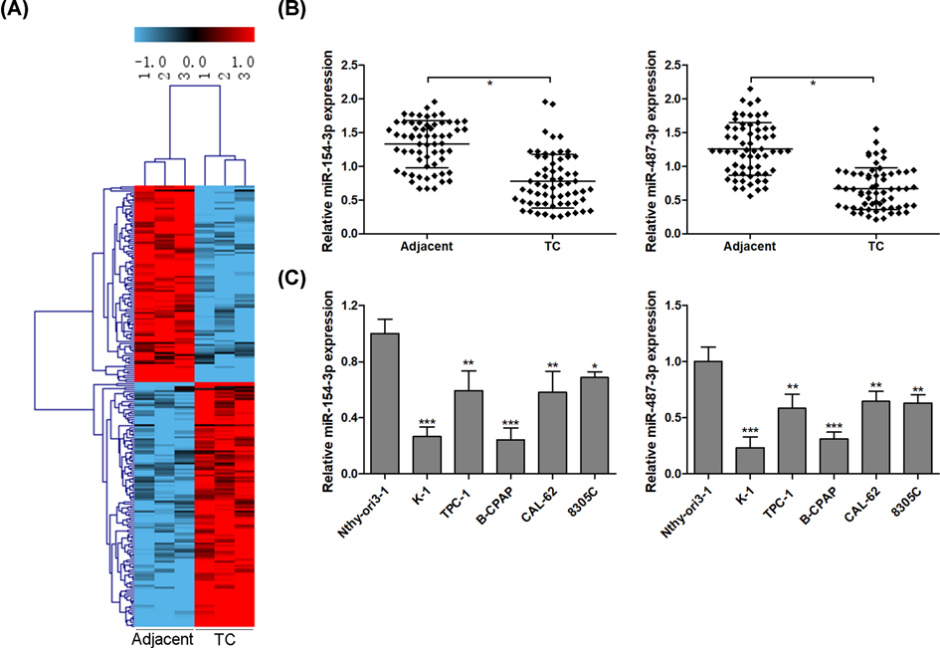
miR-154-3p and miR-487-3p are down-regulated in thyroid cancer tissues Using miRs microarray analysis, miRs expression profile is analyzed in three pairs of thyroid cancer and adjacent non-tumor tissues, and a total of 220 miRs are significantly abnormally expressed in thyroid cancer specimen compared with adjacent non-tumor tissues (**A**). The expression levels of miR-154-3p and miR-487-3p are detected using RT-qPCR in 63 pairs of thyroid cancer and adjacent non-tumor tissues (**B**) and thyroid cancer cell lines (**C**); * *P* < 0.05; ** *P* < 0.01; *** *P* < 0.001.

Based on |Log_2_fold change| ≥ 1, *P* < 0.001 and FDR ≤ 0.001, 220 differentially expressed miRs, including 98 down-regulated and 122 up-regulated miRs, were observed in three thyroid cancer tissues compared with corresponding non-tumor specimens using miR microarray (Figure 1A). The top 2 differentially expressed miRs were miR-154-3p and miR-487-3p with the fold change (tumor/adjacent) −5.02 and −4.91, respectively. RT-qPCR assays were performed to validate the expression levels of miR-154-3p and miR-487-3p in 63 pairs of thyroid cancer tissues and adjacent non-tumor tissues, and the results corroborated that the expression levels of miR-154-3p and miR-487-3p were significantly lower in thyroid cancer tissues than those of in the adjacent non-tumor tissues (Figure 1B). Furthermore, the expression levels of miR-154-3p and miR-487-3p in thyroid cancer cell lines were consistent with the results from thyroid cancer tissues (Figure 1C).

### miR-154-3p and miR-487-3p are associated with poor prognosis in thyroid cancer patients

**Figure 2:**
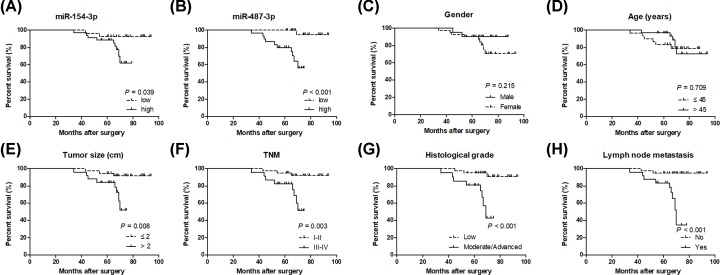
miR-154-3p and miR-487-3p are associated with poor prognosis in thyroid cancer patients miR-154-3p (**A**) and miR-487-3p (**B**) with low expression in patients with thyroid cancer had a shorter overall survival. The association between overall survival and gender (**C**), age (**D**), tumor size (**E**), TNM stage (**F**), histological grade (**G**) or lymph node metastasis (**H**) in thyroid cancer patients is evaluated using the Kaplan–Meier method with the log-rank test.

Clinicopathological data indicated that low expression of miR-154-3p or miR-487-3p was significantly correlated with bigger tumor size, poor TNM stage and histological grade, and lymph node metastasis (Table 1). Interestingly, both miR-154-3p and miR-487-3p with low expression in patients with thyroid cancer had a shorter overall survival (Figure 2A,B). Gender and age had no significant correlation with overall survival in patients with thyroid cancer (Figure 2C,D). Bigger tumor size, poor TNM stage and histological grade, and lymph node metastasis were significantly associated with poor prognosis in patients with thyroid cancer (Figure 2E–H).

**Table 1:** Correlation between clinicopathological variables and the expression of miR-154-3p and miR-487-3p in thyroid cancer tissues

Variables	*n*	miR-154-3p		*P*-value	miR-487-3p		*P*-value
		Low (*n* = 28)	High (*n* = 35)		Low (*n* = 33)	High (*n* = 30)	
**Gender**				0.151			0.593
Male	21	12	9		10	11	
Female	42	16	26		23	19	
**Age (years)**				0.735			0.516
≤45	30	14	16		17	13	
>45	33	14	19		16	17	
**Tumor size (cm)**				0.011			0.011
≤2	38	12	26		15	23	
>2	25	16	9		18	7	
**TNM**				0.012			0.009
I-II	40	13	27		16	24	
III-IV	23	15	8		17	6	
**Histological grade**				< 0.001			0.007
Low	42	12	30		17	25	
Moderate/Advanced	21	16	5		16	5	
**Lymph node metastasis**				0.001			0.021
No	39	11	28		16	23	
Yes	24	17	7		17	7	

### RHOA and ROCK1 are up-regulated in thyroid cancer tissues

**Figure 3:**
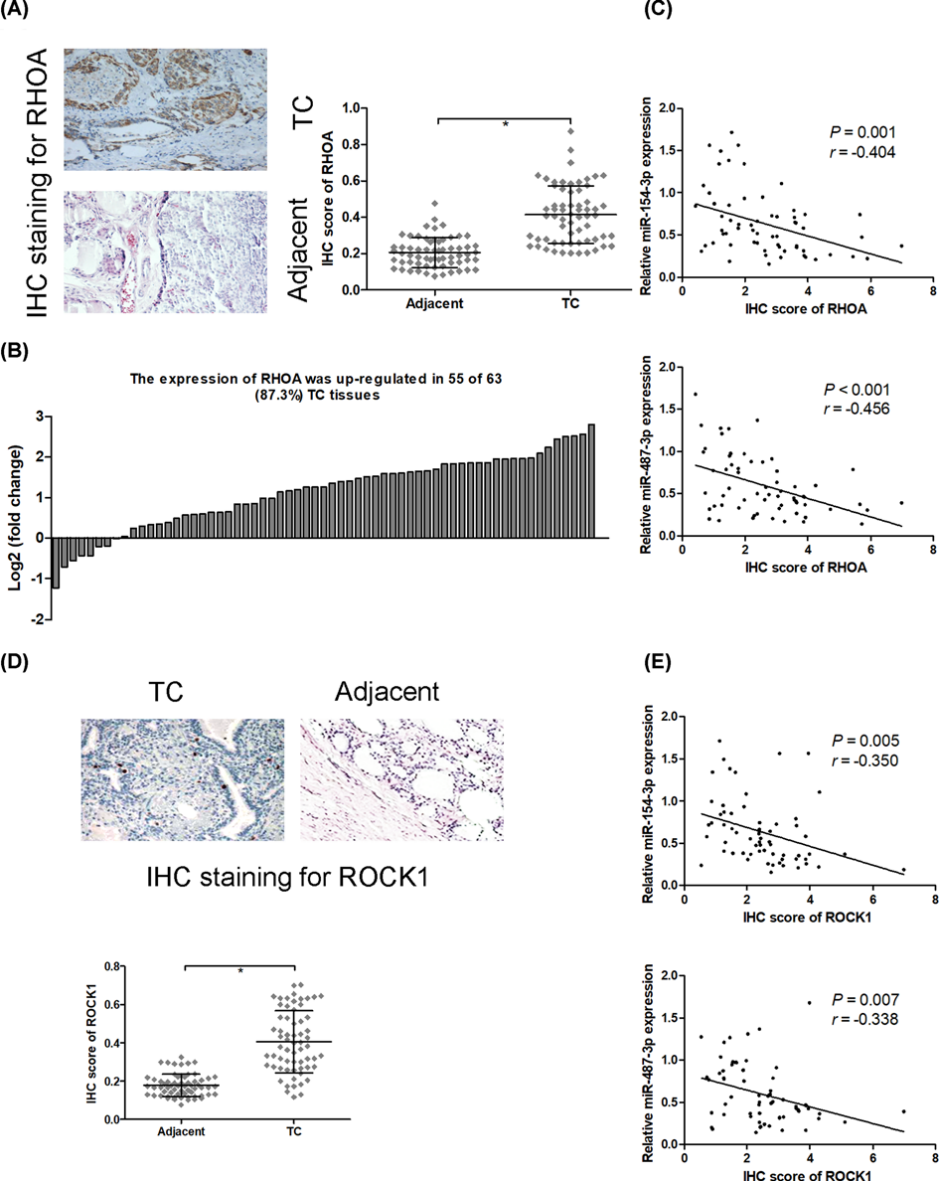
RHOA and ROCK1 are up-regulated in thyroid cancer tissues IHC staining is used to evaluate RHOA protein expression in thyroid cancer tissues and adjacent non-tumor tissues (**A**), and the protein expression of RHOA was up-regulated in 55 of 63 (87.3%) thyroid cancer tissues (**B**). Spearman’s rank analysis was used to identify the correlation between the expression levels of RHOA and miR-154-3p or miR-487-3p in thyroid cancer tissues (**C**). IHC staining is used to evaluate ROCK1 protein expression in thyroid cancer tissues and adjacent non-tumor tissues (**D**). Spearman’s rank analysis was used to identify the correlation between the expression levels of ROCK1 and miR-154-3p or miR-487-3p in thyroid cancer tissues (**E**); * *P* < 0.05.

IHC staining suggested that a significant increase RHOA protein expression was observed in thyroid cancer compared with adjacent non-tumor tissues, and the protein expression of RHOA was up-regulated in 55 of 63 (87.3%) thyroid cancer tissues (Figure 3A,B). In addition, the result of correlation analysis showed that the protein expression of RHOA was significantly inversely correlated with miR-154-3p (*r* = −0.404; *P* = 0.001) and miR-487-3p (*r* = −0.456; *P* < 0.001) expression in thyroid cancer tissues (Figure 3C). IHC staining results also exhibited that ROCK1 protein expression was significantly elevated in thyroid cancer tissues compared with adjacent non-tumor tissues (Figure 3D). Correlation analysis revealed that the protein expression of ROCK1 was significantly negatively correlated with miR-154-3p (*r* = −0.350; *P* = 0.005) and miR-487-3p (*r* = −0.338; *P* = 0.007) expression in thyroid cancer tissues (Figure 3E).

### RHOA is a direct target of miR-154-3p and miR-487-3p

**Figure 4:**
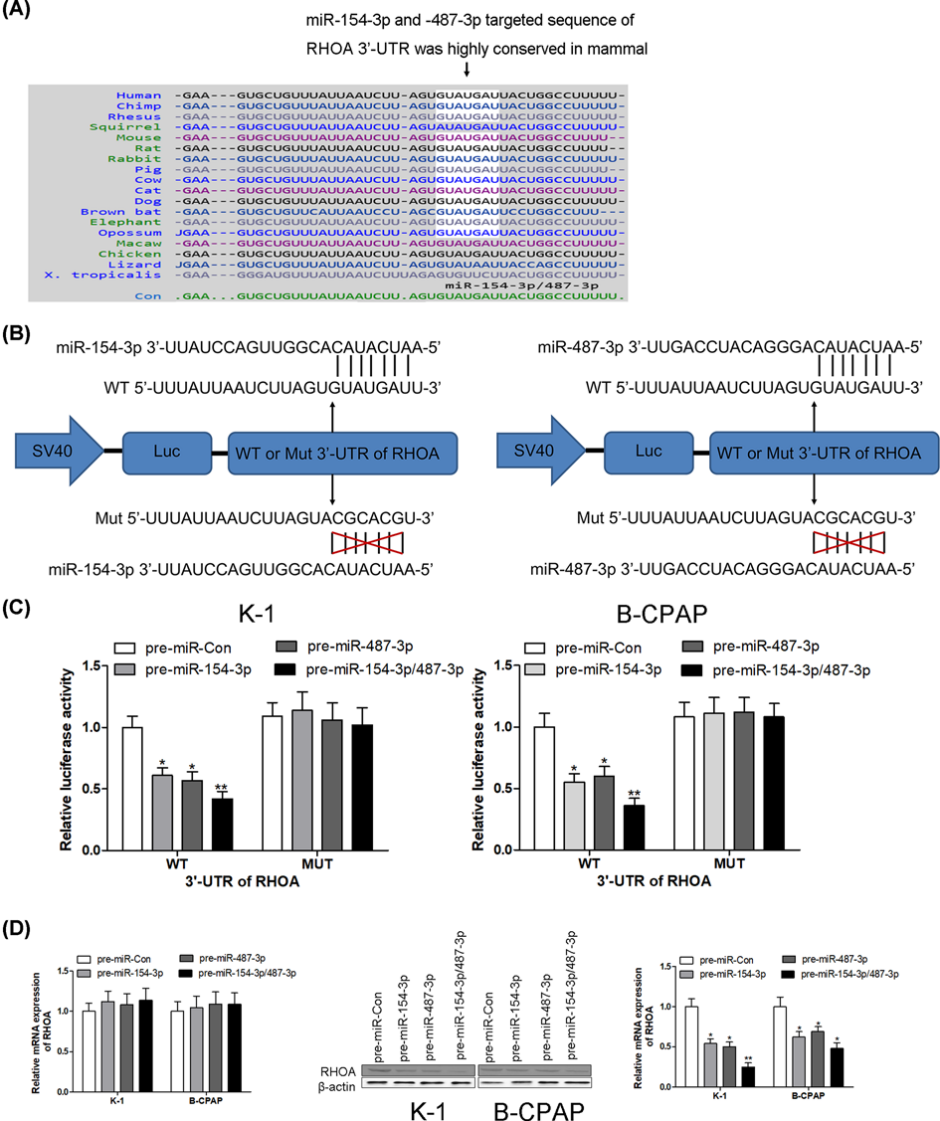
RHOA is a direct target of miR-154-3p and miR-487-3p Using on-line bioinformatics algorithm (TargetScan, http://www.targetscan.org/), the conserved sequence of binding sites between miR-154-3p/-487-3p and RHOA was shown across various species (**A**); miR-154-3p and miR-487-3p are identified as candidate miRNAs targeting to RHOA (**B**). The association between miR-154-3p/487-3p and RHOA is further examined by evaluating luciferase activity in human thyroid cancer cell lines K-1 and B-CPAP (**C**). After transfection with pre-miR-Con, pre-miR-154-3p, pre-miR-487-3p or pre-miR-154-3p/487-3p into K-1 and B-CPAP cells, the mRNA and protein levels of RHOA are detected using RT-PCR and Western blotting, respectively (**D**); * *P* < 0.05; ** *P* < 0.01.

Using on-line bioinformatics algorithm (TargetScan, http://www.targetscan.org/), miR-154-3p and miR-487-3p were identified as candidate miRNAs targeting to RHOA. The binding sites between miR-154-3p/-487-3p and RHOA were highly conserved across many species (Figure 4A). As shown in Figure 4B, both miR-154-3p and miR-487-3p could bind with the 3′-UTR of RHOA and share sequence homology. The association between miR-154-3p/487-3p and RHOA was further examined by evaluating luciferase activity in human thyroid cancer cell lines K-1 and B-CPAP. In our further experiments, miR-154-3p or miR-487-3p gain-of-function was achieved by transfecting with pre-miR-154-3p or pre-miR-487-3p, which are synthetic RNA oligonucleotides that mimic the miR-154-3p and miR-487-3p precursors. As expected, the luciferase activity was significantly declined in K-1 and B-CPAP cells after transfection with pre-miR-154-3p or pre-miR-487-3p compared with pre-miR-Con group. Intriguingly, the inhibition efficiency on luciferase activity was enhanced after co-transfection with pre-miR-154-3p and pre-miR-487-3p (Figure 4C). However, after transfection with pre-miR-154-3p, pre-miR-487-3p or pre-miR-154-3p/487-3p, the luciferase activity had no obvious change in K-1 and B-CPAP cells containing Mut 3′-UTR of RHOA (Figure 4C). After transfection with pre-miR-Con, pre-miR-154-3p, pre-miR-487-3p or pre-miR-154-3p/487-3p into K-1 and B-CPAP cells, the mRNA levels of RHOA had no significant difference in those groups; however, the protein levels of RHOA were dramatically reduced in K-1 and B-CPAP cells when transfection with pre-miR-154-3p, pre-miR-487-3p or pre-miR-154-3p/487-3p compared with the control group (Figure 4D). These results suggest that miR-154-3p and miR-487-3p specifically repress RHOA expression at the post-transcriptional level, but not mRNA levels, in human thyroid cancer, which represents the canonical regulatory mechanism of miRs in mammal.

### miR-154-3p and miR-487-3p inhibit cell proliferation and induce apoptosis via targeting RHOA

**Figure 5:**
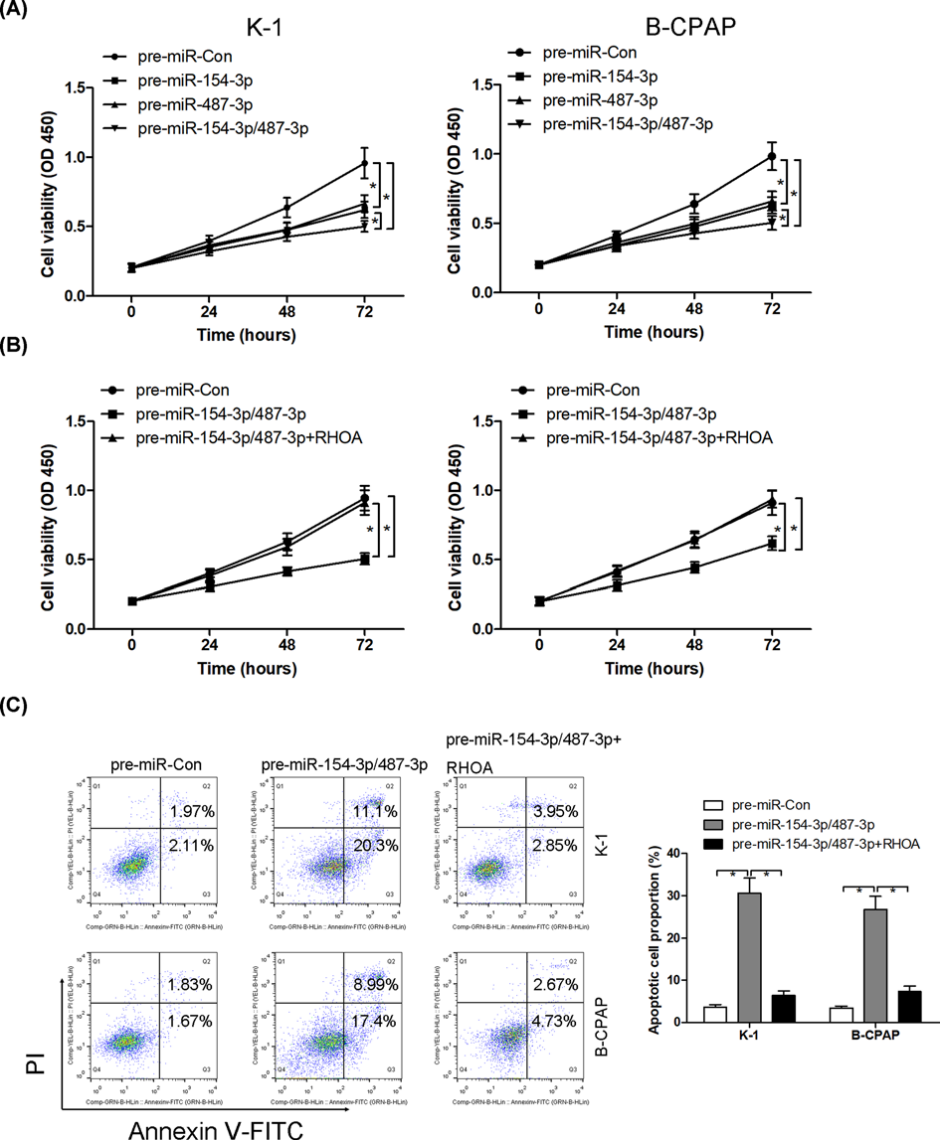
miR-154-3p and miR-487-3p inhibit cell proliferation and induce apoptosis via targeting RHOA After transfection with pre-miR-Con, pre-miR-154-3p, pre-miR-487-3p or pre-miR-154-3p/487-3p into K-1 and B-CPAP cells, cell viability is analyzed using CCK8 assays (**A**). After transfection with pre-miR-Con, pre-miR-154-3p/487-3p and pre-miR-154-3p/487-3p combined with RHOA overexpressed vectors into K-1 and B-CPAP cells, cell viability and apoptosis are analyzed using CCK8 (**B**) and Annexin V-FITC double staining (**C**), respectively; * *P* < 0.05.

To determine the biological consequences of miR-154-3p and miR-487-3p in thyroid cancer cell lines K-1 and B-CPAP, pre-miR-154-3p, pre-miR-487-3p or pre-miR-154-3p/487-3p was transfected into K-1 and B-CPAP cells, and cell proliferation and apoptosis were evaluated using CCK8 and Annexin V-FITC double staining, respectively. miR-154-3p and miR-487-3p gain-of-function could individually inhibit cell proliferation, the simultaneous introduction of both miR-154-3p and miR-487-3p synergistically suppressed cell proliferation in K-1 and B-CPAP cells (Figure 5A). Meanwhile, co-transfection with pre-miR-154-3p/487-3p and vector-RHOA into K-1 and B-CPAP cells, overexpression of RHOA reversed the anti-proliferative and pro-apoptotic effect of pre-miR-154-3p/487-3p (Figure 5B,C).

### miR-154-3p and miR-487-3p repress thyroid cancer K-1 cell growth *in vivo*

**Figure 6:**
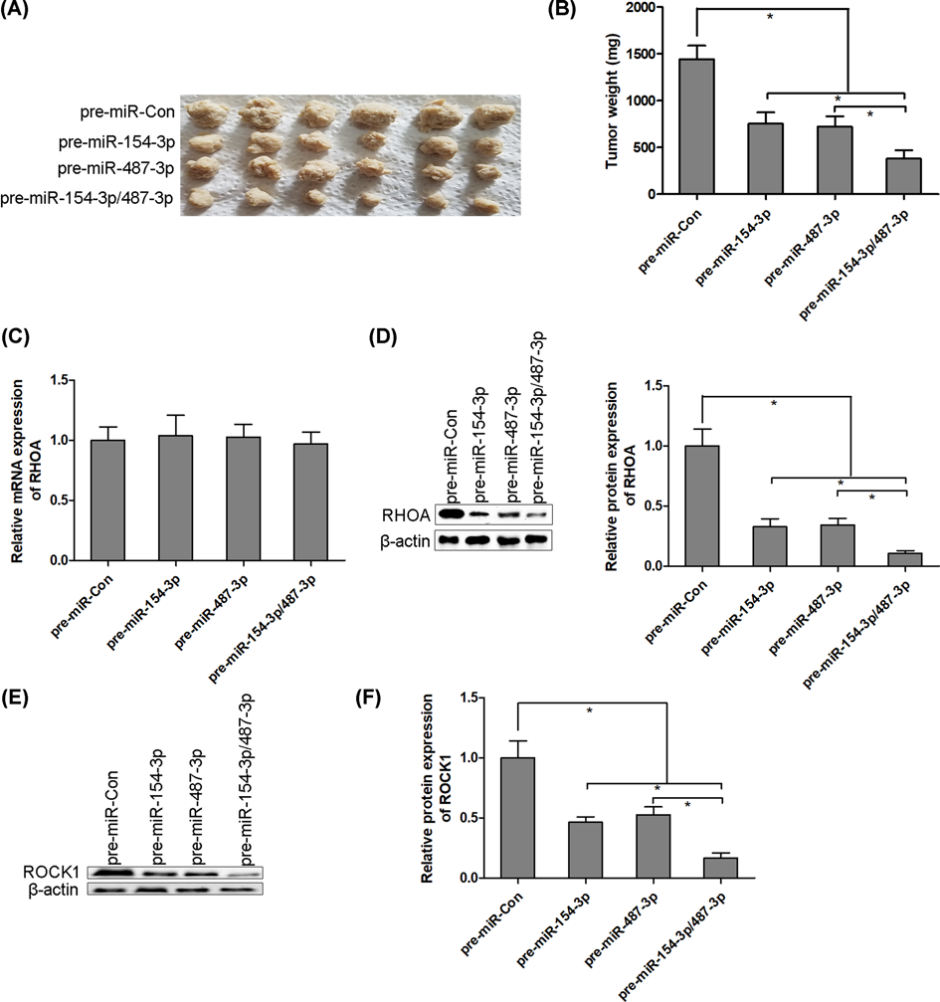
miR-154-3p and miR-487-3p repress thyroid cancer K-1 cell growth *in vivo* Thyroid cancer K-1 cells (1 × 10^7^ cells per 0.1 ml) were established to steadily express miR-154-3p, miR-487-3p or miR-154-3p/487-3p. K-1 cells were implanted subcutaneously into 4-week-old BALB/c nude mice, and tumor growth was evaluated at week 4 after K-1 cells implantation (**A** and **B**). The mRNA and protein levels of RHOA are detected using RT-PCR and Western blotting, respectively, in solid tumors of K-1 cells transplanted nude mice (**C** and **D**). The protein levels of ROCK1 are detected using Western blotting in solid tumors of K-1 cells transplanted nude mice (**E** and **F**); * *P* < 0.05.

We next determined the anti-proliferative effect of miR-154-3p and miR-487-3p in nude-mouse transplanted tumor model. Thyroid cancer K-1 cells (1 × 10^7^ cells per 0.1 ml) were established to steadily express miR-154-3p, miR-487-3p or miR-154-3p/487-3p. K-1 cells were implanted subcutaneously into 4-week-old BALB/c nude mice, and tumor growth was evaluated at week 4 after K-1 cells implantation. The tumor weight was significantly inhibited in miR-154-3p or miR-487-3p overexpressed mice compared with the control group. Moreover, miR-154-3p and miR-487-3p synergistically suppressed K-1 cells growth *in vivo* (Figure 6A,B). We also found that miR-154-3p and miR-487-3p synergistically inhibited RHOA protein expression, but not mRNA levels, in solid tumors of K-1 cells transplanted nude mice (Figure 6C,D). The similar results of miR-154-3p and miR-487-3p on ROCK1 protein expression had been observed in solid tumors of K-1 cells transplanted nude mice (Figure 6E,F).

## Discussion

Recently, RHOA is frequently reported as an oncogenic gene implicating in the initiation and progression of malignant tumors via exacerbating cell migration and invasion [6,8,19,20]. RHOA is widespreadly overexpressed in prostate cancer, cervical cancer and colorectal cancer, and associated with cancer metastasis [21–23]. Thus, we speculate that RHOA may serve as a particularly feasible molecular target for the treatment of malignant tumors. However, very little is known about the underlying molecular mechanism and the expression of RHOA in thyroid cancer. Thus, we aimed to investigate the malignant properties of RHOA in thyroid cancer, which might provide a novel therapeutic target in thyroid cancer.

In the present study, we found that RHOA protein expression was significantly elevated in 63 thyroid cancer tissues compared with adjacent non-tumor tissues. Bioinformatics algorithm revealed that RHOA is a direct target of miR-154-3p and miR-487-3p, which are belonged to a families of miRNAs. The expression of miR-154-3p is found to be down-regulated in breast cancer [24]. Interestingly, the protein expression of RHOA was significantly negatively correlated with miR-154-3p and miR-487-3p expression in 63 thyroid cancer tissues.

miRs play crucial roles in modulating gene expression via post-transcriptional repression [25,26]. In fact, single miR can modulate multiple target genes, in contrast with that, one gene can be regulated by multiple miRs [25,26]. For example, miR-143 and miR-145 gain-of-function synergistically suppress cell proliferation and invasion in breast cancer through the repression of ERBB3 protein expression [25]. miR-148-3p and miR-152-3p realize synergistic effect to inhibit cell proliferation and induce apoptosis in prostate cancer cells [26]. In thyroid cancer, we discovered a collaborative mechanism of miRs that miR-154-3p and miR-487-3p tend to function as tumor suppressors in human thyroid cancer via post-transcriptional repression of RHOA protein expression, but not mRNA levels.

Our findings demonstrated that miR-154-3p and miR-487-3p were significantly decreased in 63 thyroid cancer tissues and cell lines compared with those in paired non-tumor tissues and normal thyroid follicular epithelial cells. Low expression levels of miR-154-3p and miR-487-3p significantly correlated with tumor size, TNM stage, histological grade, lymph node metastasis and shorter overall survival in patients with thyroid cancer. Furthermore, we experimentally validated that miR-154-3p and miR-487-3p synergistically blocked thyroid cancer cell growth *in vitro* and *in vivo*. However, the anti-proliferative and pro-apoptotic activities of miR-154-3p/487-3p were neutralized by RHOA overexpressed vectors. Our present findings expound a novel signal cascade employing miR-154-3p/487-3p and RHOA to fine-tune thyroid cancer cell proliferation and apoptosis. Therefore, we corroborate that the suppression of RHOA by miR-154-3p/487-3p may be a valuable therapeutic target for impeding thyroid cancer progression.

One primary goal of the present study was to explore whether miR-154-3p or miR-487-3p performs its function individually or synergistically. First, we found that miR-154-3p or miR-487-3p are grouped into families, which contribute to have sequence homology and possess the same seed sequence to bind with target gene [25]. Usually, miRs family members are often thought to have extensively overlapping targets [25]. Experimental measurements strongly supported the hypothesis that miR-154-3p and miR-487-3p exhibited a synergistic repression of luciferase activity and RHOA protein expression. In addition, miR-154-3p and miR-487-3p showed a cooperative effect to inhibit thyroid cancer cell growth *in vitro* and *in vivo*. Our results suggest that multiple miRs collaborate to suppress the same target gene that may be more efficient and potent.

However, some limitations existed in the present study. First, the number of clinical specimen is too small, which may influence the reliability of the present conclusions. Second, the roles of miR-154-3p, miR-487-3p and RHOA on cell migration and invasion had not been investigated in the present study.

In conclusion, our findings highlighted a critical anti-neoplastic role of miR-143 and miR-145 and also showed that RHOA as an oncogene could be post-transcriptionally repressed by miR-143 and miR-145 in thyroid cancer progression.

## Abbreviations

PBS: phosphate-buffered saline
RHOA: Ras homolog family member A
ROCK: Rho-associated protein kinase
